# Injectable Thermosensitive Hydrogels for a Sustained Release of Iron Nanochelators

**DOI:** 10.1002/advs.202200872

**Published:** 2022-03-27

**Authors:** Seung Hun Park, Richard S. Kim, Wesley R. Stiles, Minjoo Jo, Lingxue Zeng, Sunghoon Rho, Yoonji Baek, Jonghan Kim, Moon Suk Kim, Homan Kang, Hak Soo Choi

**Affiliations:** ^1^ Gordon Center for Medical Imaging Department of Radiology Massachusetts General Hospital and Harvard Medical School Boston MA 02114 USA; ^2^ Department of Molecular Science and Technology Ajou University Suwon 16499 South Korea; ^3^ Department of Biomedical & Nutritional Sciences Zuckerberg College of Health Sciences University of Massachusetts Lowell MA 01854 USA

**Keywords:** chelation therapy, deferoxamine, hydrogel, iron chelator, sustained release

## Abstract

Deferoxamine (DFO) is an FDA‐approved iron‐chelating agent which shows good therapeutic efficacy, however, its short blood half‐life presents challenges such as the need for repeated injections or continuous infusions. Considering the lifelong need of chelating agents for iron overload patients, a sustained‐release formulation that can reduce the number of chelator administrations is essential. Here, injectable hydrogel formulations prepared by integrating crosslinked hyaluronic acid into Pluronic F127 for an extended release of DFO nanochelators are reported. The subcutaneously injected hydrogel shows a thermosensitive sol–gel transition at physiological body temperature and provides a prolonged release of renal clearable nanochelators over 2 weeks, resulting in a half‐life 47‐fold longer than that of the nanochelator alone. In addition, no chronic toxicity of the nanochelator‐loaded hydrogel is confirmed by biochemical and histological analyses. This injectable hydrogel formulation with DFO nanochelators has the potential to be a promising formulation for the treatment of iron overload disorders.

## Graphical abstract

Injectable hydrogel formulation prepared by integrating crosslinked hyaluronic acid into Pluronic F127 shows a thermosensitive sol–gel transition at body temperature and provides a prolonged release of renal clearable iron nanochelators over 2 weeks, resulting in a half‐life 47‐fold longer than that of the nanochelator alone.

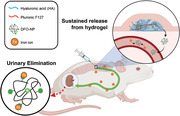

## 1 Introduction

Iron is an essential metal yet high iron stores are toxic due to metal‐induced oxidative stress that promotes organ damage including heart failure, liver cirrhosis, diabetes, and neurodegenerative diseases.^[^ ^1^ ^]^ Primary iron overload (hereditary hemochromatosis) is one of the most common genetic diseases, affecting 1 million people worldwide, mainly the Caucasian population. Secondary iron overload can occur in patients with hemoglobinopathies, such as thalassemia major, sickle cell anemia, aplastic anemia, myelofibrosis, myelodysplastic syndrome, and Diamond–Blackfan anemia because they require chronic blood transfusions.^[^ ^2^ ^]^ Since there is no recognized active pathway of iron excretion,^[^ ^3^ ^]^ chelation therapy has been widely used to improve disease conditions in patients with iron overload, especially transfusion‐associated iron accumulation.^[^ ^4^ ^]^

There are three small molecule‐based iron chelators currently in use: deferoxamine (DFO), deferiprone (DFP), and deferasirox (DFX). In particular, DFO has shown good therapeutic efficacy since being approved by the U.S. FDA in 1968. However, the very short half‐life (e.g., 5–15 min in rodents^[^ ^5^ ^]^) of DFO requires repeated injections or continuous infusions which considerably reduce patient compliance and their quality of life.^[^ ^6^ ^]^ The other two chelators, DFP and DFX, are orally administrable and therefore overcome this compliance issue.^[^ ^7^ ^]^ However, DFP and DFX cause significant adverse effects including gastrointestinal tract bleeding, agranulocytosis, neutropenia, thrombocytopenia, hepatic fibrosis, and kidney failure.^[^ ^7^ ^]^ Although other orally active iron chelators, like derivatives of desferrithiocin, are in clinical trials or preclinical phases, they have nephrotoxic effects.^[^ ^8^ ^]^ Several studies have attempted to improve the pharmacokinetics/pharmacodynamics (PK/PD) and tissue distribution, as well as reduce the side effects of DFO exploiting macromolecules by conjugating them to biocompatible polymers (i.e., dextranDFO, pentastarch‐DFO, hydroxyethyl starch‐DFO, and dendritic polyglycerol) (Table S1, Supporting Information).^[^ ^9^ ^]^ Previously, as part of these efforts, we developed nanochelators (DFO‐NPs) that have favorable PK/PD and the capability of fast iron elimination to urine.^[^ ^10^ ^]^ DFO‐NP showed no acute toxicity after intravenous injection with a high dose (1500 mg kg^−1^). However, repeated administration of DFO‐NP is necessary because iron overload patients require lifelong treatment.

Currently, several sustained drug release systems have been developed such as hydrogels, patches, implantable drug devices, and infusion pumps, which significantly improve the compliance and adherence issues of patients.^[^ ^11^ ^]^ Especially, injectable thermosensitive hydrogels have been frequently used for drug delivery because they do not require additional chemical reactions and external stresses (e.g., light or pressure) to crosslink.^[^ ^12^ ^]^ However, many polymeric hydrogels show potential toxicity. Thus, it is necessary to develop a hydrogel formulation with FDA‐approved materials to accelerate the progress of clinical trials.^[^ ^13^ ^]^

**Figure 1:**
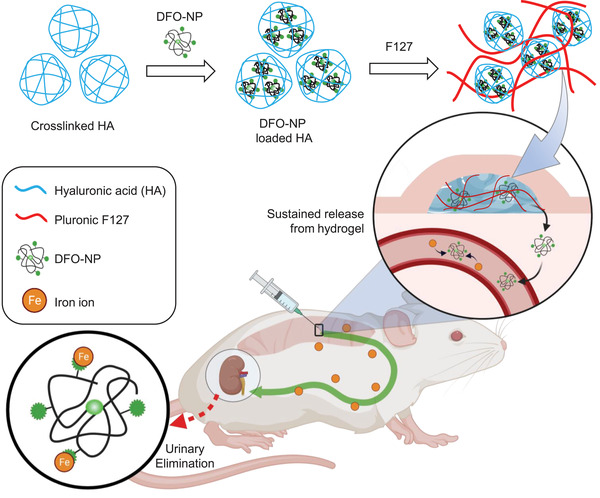
Schematic diagram of DFO‐NP loaded injectable hydrogels. DFO‐NPs were loaded in crosslinked‐hyaluronic acid (xHA), and F127 was formulated as an outer hydrogel. Sustained‐release of DFO‐NPs from the hydrogel significantly improves their pharmacokinetics and minimized off‐target tissue distribution.

Here, we report injectable thermosensitive hydrogels for the long‐term release of DFO‐NPs for iron chelation therapy. The injectable hydrogel was prepared by integrating crosslinked‐hyaluronic acid (xHA) into Pluronic F127, both FDA‐approved, to load a high dose of DFO‐NP and to diminish the initial burst release. The hydrogel showed thermosensitive rheological properties, and its release kinetics were evaluated longitudinally by using a NIR fluorescence imaging system (**Figure** **1**). This sustained release hydrogel formulation could potentially improve the therapeutic efficacy while minimizing the toxicity by offering a long‐term release of iron nanochelators with a short‐term residence in non‐target tissues, which is beyond the capability of current small molecule chelators. Considering the need for lifelong administrations of chelators in patients with iron overload, hydrogel‐based nanochelators offer significant advantages over the current chelation therapies.^[^ ^10^ ^]^

## 2 Results and Discussion

### 2.1 Synthesis and Characterization of DFO‐NP

Prior to designing the injectable hydrogel formulation, the DFO‐NP was prepared as previously reported. Briefly, zwitterionic NIR fluorophore (ZW800‐1C) was conjugated to *ε*‐poly‐l‐lysine (ZW‐EPL^+^) for monitoring of the in vivo behavior of DFO‐NP. All of the primary amines of ZW‐EPL^+^ were succinylated (ZW‐EPL^−^) to convert the side chains into negatively charged carboxylate groups and hence enable DFO conjugation via amide bonding on ZW‐EPL^−^ (DFO‐NP, Figure S1, Supporting Information). Each product was analyzed and the number of DFOs on DFO‐NP was quantified to be ≈1.0 by nuclear magnetic resonance (NMR) spectroscopy (Figure S2, Supporting Information). The peaks at 4.12 ppm (position *α*′) and 3.6 ppm (position e,l,s) correspond to the protons of EPL and DFO, respectively. The hydrodynamic diameter (HD) was analyzed by high‐performance liquid chromatography (HPLC) with a size exclusion chromatography column. The HD of an NP is one of the key factors in predicting its clearance route, and HDs smaller than the renal threshold (6–8 nm) are preferred for renal clearance.^[^ ^14^ ^]^ Indeed, the HD of DFO‐NP was calculated to be 5.2 nm when compared to the calibration curve of a known standard protein set, which suggested DFO‐NP can be renally clearable (Figure S3, Supporting Information). In addition, the purity of DFO‐NP was confirmed to be 96% which suggests the absence of impurities such as unconjugated DFO and ZW800‐1C. The optical properties and iron‐binding efficacy of DFO‐NP were evaluated by spectrophotometry. The maximum wavelengths of absorption and fluorescence emission for DFO‐NP were 760 and 780 nm, respectively, (Figure S4a, Supporting Information) which allow the DFO‐NP to be detected in a NIR channel with minimal tissue scattering and autofluorescence. The iron‐binding efficacy of DFO‐NP was confirmed in an in vitro iron‐binding assay using ferric chloride (FeCl_3_). Since the complex of DFO and Fe^3+^ ion absorbs wavelengths of 430 nm, the chelation of DFO‐NP with iron could be easily observed by measuring UV–vis absorbance.^[^ ^10^, ^15^ ^]^ The absorption value at 430 nm increased as the 5 µL FeCl_3_ solutions (4 × 10 ^−3^ m) were sequentially added to the DFO‐NP solution (1000 µL, 100 × 10^−6^ m) (Figure S4b, Supporting Information). After adding 25 µL of FeCl_3_ solution, which is equimolar to the DFO‐NP solution, it was shown that the increase of absorption at 430 nm was reduced (Figure S4c, Supporting Information). These results indicate that the stoichiometry value of DFO‐NP is ≈1.0 due to DFO forming a monodentate complex with Fe^3+^ ions, which is consistent with the ^1^H‐NMR result for proton quantification.

Next, an in vitro cellular uptake experiment was performed to confirm the nonsticky property of DFO‐NP in cells (Figure S5a, Supporting Information). NIH3T3 and NCL‐H23 (H23) cells were selected as representative normal and cancerous cells, respectively, and positively charged ZW‐EPL^+^ was used as a positive control. In fluorescence microscope images, ZW‐EPL^+^ treated cells showed strong NIR fluorescence signals after 2 h, while DFO‐NP treated cells exhibited no signal, suggesting that the negatively charged surface of DFO‐NP can minimize nonspecific cell uptake. In addition, the cytotoxicity of DFO‐NP was tested in NIH3T3 cells by incubating with various concentrations of DFO‐NP (1, 5, 10, 50, and 100 × 10^−6^ m) for 24 h, showing there is no cytotoxicity associated with DFO‐NP (Figure S5b, Supporting Information).

### 2.2 Preparation and Characterization of the Injectable Hydrogels

Pluronic F127 and HA were selected as materials for the injectable hydrogel formulation because they are FDA‐approved and widely used in various fields including drug delivery systems.^[^ ^16^ ^]^ Pluronic is a non‐ionic triblock copolymer, consisting of a central hydrophobic chain of polyoxypropylene flanked by two hydrophilic chains of polyoxyethylene, which has a unique thermosensitive sol–gel transition facilitated by a micellar mechanism when concentrated in water. HA is used clinically as a dermal filler and drug delivery vehicle due to its superior biocompatibility, minimal toxicity, and low inflammatory properties.^[^ ^17^ ^]^ Thus, we implement HA into the hydrogel to control the initial burst release of F127. To start the hydrogel formulation process, HA was crosslinked with 1,4‐butanediol diglycidyl ether (BDDE) which has been widely used in the manufacturing of dermal fillers (Figure S6, Supporting Information).^[^ ^18^ ^]^ Since crude xHA is barely injectable, it was crushed through 23–25 gauge needles by applying pressure,^[^ ^19^ ^]^ resulting in the crushed xHA particulates (HAPs) which could be injected via syringe with a 23G needle. The HAPs were lyophilized and subsequently immersed into DFO‐NP solution to load the DFO‐NPs into HAPs. Dried DFO‐NP loaded HAPs were added into Pluronic F127 solution for the final hydrogel formulation. The final weight percentages of DFO‐NP, HAP, and Pluronic F127 are 30, 7, and 30 (denoted as DFO‐NP/HA/F127; 30/7/30%), and the final concentration of DFO‐NP in the formulation was 40 × 10^−3^ m. To compare the effects of hybrid HA and F127 hydrogel, DFO‐NP/HA; 30/7% and DFO‐NP/F127; 30/30% were prepared as controls. All prepared formulations were injectable using a syringe with a 23G needle.

**Figure 2:**
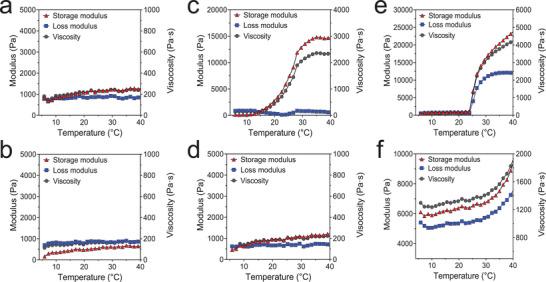
Rheological properties of hydrogel formulations. A) Crosslinked hyaluronic acid (HA; 7%) and B) DFO‐NP loaded HA (DFO‐NP/HA; 30/7%), C) Pluronic F127 (F127; 30%) and D) DFO‐NP loaded F127 (DFO‐NP/F127; 30/30%), E) HA and F127 integrated hydrogel (HA/F127 7/30%), and F) DFO‐NP loaded HA/F127 (DFO‐NP/HA/F127; 30/7/30%).

To evaluate the thermosensitivity of prepared hydrogels, rheological properties such as loss modulus, storage modulus, and viscosity were measured over a temperature ramp from 4 to 40 °C (**Figure** **2**). Since the high content of drugs in thermosensitive hydrogels generally suppresses their thermo‐gelling property,^[^ ^20^ ^]^ each corresponding hydrogel without DFO‐NPs (HA; 7%, F127; 30%, and HA/F127; 7/30%) were also tested to confirm the effect of DFO‐NPs on gelation. HA and DFO‐NP/HA showed no significant change in rheological properties as the temperature ramped up (Figure 2a,b), and the storage and loss moduli stayed below 1000 Pa. The F127 showed a continuous increase of storage modulus and viscosity in a temperature range from 14 to 32 °C which indicates a thermosensitive sol‐gel transition at 15 °C (Figure 2c,d). In contrast, no thermo‐responsive behavior in the DFO‐NP/F127 was observed indicating that the high content of DFO‐NP interrupted the hydrophobic aggregation of Pluronic micelles. Interestingly, both HA/F127 and DFO‐NP/HA/F127 exhibited thermo‐gelling properties (Figure 2e,f). In HA/F127, all rheological parameters significantly increased above 25 °C which is likely attributed to a reinforcement by the HAP network. Despite the high content of DFO‐NP, the moduli and viscosity values for DFO‐NP/HA/F127 were higher than that of HA/F127 in the low‐temperature range (<25 °C). In addition, DFO‐NP/HA/F127 showed thermosensitive rheological changes above 30 °C and the storage modulus increased up to 8500 Pa. These results suggest that DFO‐NP/HA/F127 is not only injectable but also can form a firm hydrogel at body temperature due to high moduli.

### 2.3 In Vivo Release and Pharmacokinetics of DFO‐NP Loaded Injectable Hydrogels

**Figure 3:**
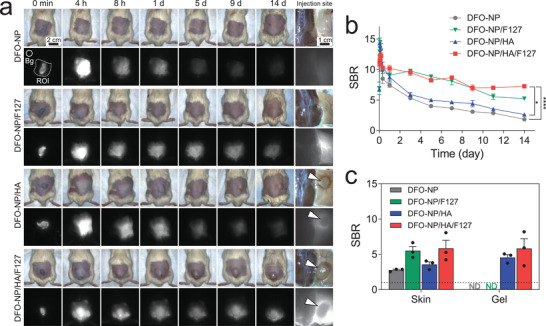
In vivo longitudinal monitoring of DFO‐NP release from various hydrogel formulations. A) Representative color and NIR fluorescence images of CD‐1 mice subcutaneously injected with DFO‐NP and DFO‐NP loaded hydrogels, such as DFO‐NP/F127, DFO‐NP/HA, and DFO‐NP/HA/F127 for up to 14 d postinjection. White arrowheads indicate remaining hydrogels on day 14. Exposure time: 50 ms. B) Longitudinal profiling of signal‐to‐background ratio (SBR) of hydrogel injected sites (region of interest; ROI) against background signal (Bg) *(n* = 3 per group, mean ± s.e.m.). C) SBR of skin and hydrogels against muscle after peeling off the skin at 14 d. *p* values < 0.05 were considered significant: *p < 0.05, ****p < 0.001.

To evaluate the sustained‐release property of the above hydrogel formulations, DFO‐NP loaded injectable formulations and DFO‐NP solution were subcutaneously injected into the back of mice. The injected dose of DFO‐NP was 125 µmol kg^−1^ in all mice. The fluorescent signal of DFO‐NP in mice was observed with a real‐time NIR fluorescence imaging system (K‐FLARE) for 14 d after injection (**Figure** **3**). The fluorescence signal in all experimental groups decreased rapidly 1 d after injection due to the initial burst release. In the DFO‐NP solution injected mice, the fluorescence signal became negligible after 5 d and almost no fluorescent signal was observed after 14 d even after peeling off the skin at the injection site. The fluorescence intensity and signal‐to‐background ratio (SBR) of DFO‐NP/F127 decreased slowly from 10 to 5 over 1 to 14 d, and there was no remaining hydrogel at the injection site. For DFO‐NP/HA, the observed fluorescence signal as well as SBR were lower than those of DFO‐NP/F127 after 1 d and decreased gradually over time. However, hydrogels were found at the injection sites after 14 d (white arrowheads in Figure 3a). The fluorescence signal in the DFO‐NP/HA/F127 group diminished more slowly than those of the DFO‐NP and DFO‐NP/HA groups, of which the SBR pattern was similar to that of the DFO‐NP/F127 group (Figure 3b). The SBR of DFO‐NP/HA/F127 was maintained at around 7.0 from 9 to 14 d, which was significantly higher than the other groups. In addition, the hydrogel still remained in the DFO‐NP/HA/F127 group, and a relatively higher fluorescence intensity was observed around the hydrogel at the injection site after 14 d (Figure 3a,b). For a more detailed comparison, we calculated the SBR of the skin and hydrogels at the injection sites after 14 d (Figure 3c).

Although the initial burst release of DFO‐NP could not be fully suppressed in the hydrogel formulations, it is worth noting that the rapid removal of nontransferrin bound iron, the predominant form of iron circulating in the blood of iron overload patients, is important to prevent recurrence of the disease. Therefore, indeed, the initial release of some portion of the nanochelators from the hydrogel would have a positive effect on the treatment of iron overload. Interestingly, F127 micelles tend to be absorbed by the skin near the injection site,^[^ ^22^ ^]^ which might explain the high fluorescence signals on the skin in the DFO‐NP/F127 and DFO‐NP/HA/F127 treatment groups (Figure 3a). In addition to higher skin SBR, the SBR of the remaining hydrogel in the DFO‐NP/HA/F127 group was higher than others suggesting that DFO‐NPs can be released when the hydrogel decomposes. Moreover, we observed the biodistribution of DFO‐NP in all experimental groups after 14 d postinjection (Figure S7, Supporting Information). In all groups, no fluorescence signal was observed in organs other than the kidney and bladder which clearly showed that our nanochelator, DFO‐NP, has exclusive urinary excretion and no off‐target potential toxicity.

**Figure 4:**
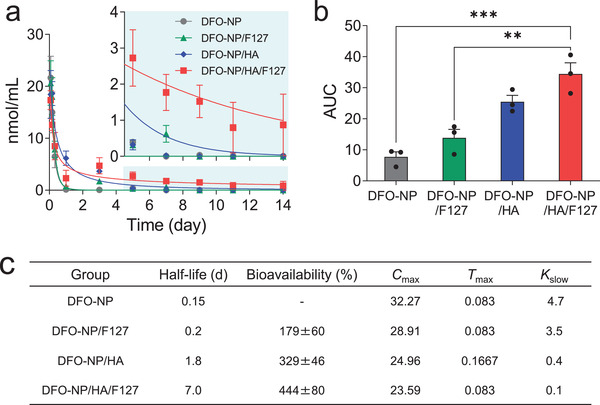
PK analysis for DFO‐NP and DFO‐NP/F127, DFO‐NP/HA, and DFO‐NP/HA/F127 hydrogels. A) Plasma concentration decay curve, B) area under the curve (AUC), and C) pharmacokinetic parameters of each sample. ^†^Relative bioavailability (AUC_Hydrogel_/AUC_DFO‐NP_ × 100) compared to DFO‐NP. The concentrations of DFO‐NP in plasma were calculated by measuring fluorescence signal intensity (*n* = 3 per group, mean ± s.e.m.). *p* values < 0.05 were considered significant: **p < 0.01, ***p < 0.005.

To further investigate the sustained release of DFO‐NP from injectable hydrogel formulations, a PK study was performed. Mouse blood samples were collected at predetermined time points throughout 14 d, and the concentration of DFO‐NP in the blood was determined at each time point by NIR fluorescent signal intensity, which was used to produce plasma concentration decay curves of the injected formulations (**Figure** **4**a; Figure S8, Supporting Information). The plasma concentrations of DFO‐NP in DFO‐NP/F127 and DFO‐NP groups were barely detectable after 3 d. This result indicates that Pluronic F127 alone is not suitable for the sustained release of DFO‐NP and that DFO‐NP absorbed into the skin with Pluronic F127 micelle is hardly released. The concentration in the DFO‐NP/HA group was well maintained until day 3. However, almost no fluorescence signal from DFO‐NP (<0.05 nmol mL^−1^) was observed after 5 d postinjection. In the DFO‐NP/HA/F127 group, the concentration of DFO‐NP exhibited a similar pattern to that of the DFO‐NP/F127 group which is mainly due to the presence of Pluronic F127 micelles in both groups. In contrast to formerly stated formulations, the signal intensity gradually decreased until 14 days but was maintained at 1 nmol mL^−1^ which is similar to the level of DFO‐NP alone when intravenously injected (2 µmol kg^−1^) at 1 h postinjection. This concentration decay suggests that DFO‐NP/HA/F127 has a sustained‐release property. For further comparison, PK parameters of the formulations were calculated from the decay curve. The area under the curve (AUC) of DFO‐NP/HA/F127 is the highest among the different formulations and is 4‐fold higher than that of DFO‐NP alone (***p < 0.001 compared to DFO‐NP and **p < 0.005 compared to DFO‐NP/F127 groups) as shown in Figure 4b. All calculated PK parameters (half‐life, bioavailability, *C*_max_, *T*_max_, and *K*_slow_) demonstrated that the PK of DFO‐NP was significantly improved when formulated with the HA/F127 hydrogel (Figure 4c). In particular, the half‐life of DFO‐NP/HA/F127 is 47, 35, and 4‐fold longer, and the bioavailability is 4, 2.5, and 1.3‐fold higher compared to DFO‐NP, DFO‐NP/HA, and DFO‐NP/F127, respectively. From these results, it was confirmed that DFO‐NP/HA/F127 successfully delayed the release of DFO‐NP.

### 2.4 In Vivo Toxicity Test of DFO‐NP Loaded Injectable Hydrogel Formulation

**Figure 5:**
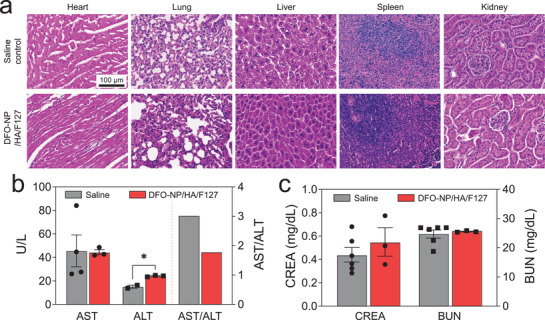
In vivo toxicity test in mice injected with saline and DFO‐NP/HA/F127 hydrogel. A) H&E staining images (20×) of heart, lung, liver, spleen, and kidney in each group. B) Serum aspartate transferase (AST), alanine transferase (ALT), and AST/ALT ratio. C) Blood urea nitrogen (BUN) and creatinine (CREA). Mice were sacrificed on 14 d postinjection for analyses. *p* values < 0.05 were considered significant: *p < 0.05.

We have proved the nontoxicity of DFO‐NP in high‐doses (1500 mg kg^−1^) via single‐dose intravenous injection in our previous paper but chronic toxicity was not yet verified. Thus, the chronic toxicity of DFO‐NP was evaluated when DFO‐NP was formulated with DFO‐NP/HA/F127 at 30/7/30% and injected subcutaneously. For histological analysis, hematoxylin and eosin (H&E) staining was performed for major organ samples (heart, lung, liver, spleen, and kidney) acquired on 14 d postinjection. (**Figure** **5**a; Figure S9, Supporting Information). In the H&E staining images of all organs, no pathological differences were observed between the saline and DFO‐NP/HA/F127 injection groups, even in the kidneys which are the most exposed organ to DFO‐NP. For further quantitative assessment for hepatotoxicity and nephrotoxicity, biochemical analyses of aspartate aminotransferase (AST), alanine transaminase (ALT), blood urea nitrogen (BUN), and creatinine (CREA) were performed. As shown in Figure 5b, there was no difference in AST levels between the saline and DFO‐NP/HA/F127 groups. ALT levels of DFO‐NP/HA/F127 were slightly higher than that of the saline control group (*p < 0.05), but within the normal range for mice (25–60 U L^−1^).^[^ ^10^ ^]^ This suggests that DFO‐NP/HA/F127 does not induce hepatotoxicity. In addition, there was no significant difference in CREA and BUN levels between the saline and DFO‐NP/HA/F127 groups (Figure 5c). This indicates that a high dose of the DFO‐NP/HA/F127 formulation does not induce any chronic toxicity.

### 2.5 In Vivo Therapeutic Efficacy of DFO‐NP Loaded Injectable Hydrogel Formulation

**Figure 6:**
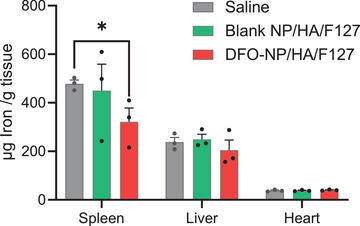
Therapeutic efficacy of DFO‐NP loaded hydrogel formulations in animal model. Male CD‐1 mice were fed with 1% carbonyl iron diet for 1 week and then treated with a single subcutaneous injection of saline, blank NP/HA/F127 (84 µmol kg^−1^ as NP), or DFO‐NP/HA/F127 (84 µmol kg^−1^ as NP). After dosing, mice were fed facility regular chow. Organs were collected 3 weeks after drug administration (*n* = 3, mean ± SEM). *p* values < 0.05 were considered significant: *p < 0.05.

To confirm the in vivo therapeutic efficacy of DFO‐NP formulations, DFO‐NP/HA/F127 hydrogel was subcutaneously injected into dietary induced iron overload (DIO) mice. For comparisons, an empty hydrogel, i.e., nanochelators without DFO (blank NP) including the same hydrogel formulation, was injected subcutaneously. Saline was used as a control. Organs (spleen, liver, and heart) were collected 3 weeks postadministration of each sample, and the iron content in the organs was measured quantitatively using ICP‐MS (**Figure** **6**). We confirmed a significant difference in iron levels in the spleen between the DFO‐NP/HA/F127 and saline groups (∆156.9 µg iron/g tissue; *p* < 0.05 compared to saline control group) and a tendency of lower iron content in the liver (∆33.0 µg iron/g tissue). The higher efficacy of iron excretion in the spleen than in the liver is likely due to greater iron overload in the spleen than in the liver.^[^ ^23^ ^]^

## 3 Conclusion

In this study, we designed and prepared injectable hydrogel formulations for the sustained release of DFO‐NP nanochelators to overcome the discomfort of repeated subcutaneous injections of DFO‐NP in patients with iron overload. We demonstrated improved PK properties and biodistribution of DFO‐NPs. It was confirmed that there was minimum to no cytotoxicity and cellular uptake as well as in vivo chronic toxicity. Also, our DFO‐NP‐loaded injectable hydrogel formulation showed thermo‐sensitivity despite the high content of DFO‐NPs, allowing the release of DFO‐NP to last for up to 14 d. Although further studies on the therapeutic efficacy of our hydrogel formulation are needed, current results show that DFO‐NP‐loaded injectable hydrogel has the potential to improve the compliance and adherence of patients with iron overload.

## 4 Experimental Section

### Materials

Epsilon‐poly‐l‐lysine (EPL; *M*_W_ ≈ 3900) was purchased from BOC Sciences (Shirly, NY). Ninhydrin agent, hyaluronic acid (100 kDa), and succinic anhydride (SA) were purchased from Acros Organics (Morris Plains, NJ). Ethyl acetate (EA), deuterium oxide (D_2_O), anhydrous dimethyl sulfoxide (DMSO), pluronic F127, 4‐(4,6‐dimethoxy‐1,3,5‐triazin‐2‐yl)‐4‐methyl‐morpholinium chloride (DMTMM), ferric chloride, sodium acetate, and sodium hydroxide were purchased from Sigma‐Aldrich (Saint Louis, MO). DFO and assay reagent kits (for aspartate aminotransferase, alanine aminotransferase, and serum creatinine) were purchased from Cayman Chemical (Ann Arbor, MI).

### Preparation of Crosslinked HA

100 mg of HA was dissolved in 600 µL of 0.3 m NaOH solution. 20 µL of 1,4‐Butanediol diglycidyl ether (BDDE) was added to the HA solution. The reaction mixture was vortexed for 1 min and incubated for 2 h at 40 °C. Then the mixture was neutralized with 0.1 m HCl to a pH of 7.0. The neutralized reaction mixture was dialyzed against deionized water (DW) using a 6–8 kDa molecular weight cutoff (MWCO) cellulose dialysis membrane to remove residual BDDE. After dialysis, the HA hydrogel was broken down to an injectable particle size using a syringe with 18–23 G needles. The injectable HA hydrogel particles (xHA) were then lyophilized.

### Preparation of DFO‐NP Loaded Injectable Hydrogels

DFO‐NP was dissolved in DW at a concentration of 1g mL^−1^. 70 mg of xHA was added to 300 µL of DFO‐NP solution to prepare DFO‐NP loaded xHA. DFO‐NP loaded xHA was lyophilized. The lyophilized DFO‐NP loaded xHA was added to 1 mL of DW (DFO‐NP/HA) or 1 mL of 30 wt% pluronic F127 solution (DFO‐NP/HA/F127) on ice before using. 300 mg of DFO‐NP was added to 1 mL of 30 wt% F127 (DFO‐NP/F127).

### Rheological Characterization of Hydrogels

The rheological properties of hydrogel formulations were analyzed using Haake Viscotester IQ Rheometer (Thermo Scientific, Germany) with a Peltier temperature‐controlled bottom plate and a 25.0 mm stainless steel parallel plate measuring system. All measurements were performed at 4–40 °C with a 0.5 mm gap. The temperature was changed by 0.03 °C s^−1^. *γ* Strain and oscillating frequency were set at 0.01 and 1 Hz, respectively.

### In Vivo Pharmacokinetics of DFO‐NPs Released from Hydrogel Formulations

Animals were housed in an AAALAC‐certified facility and were studied under the supervision of MGH IACUC in accordance with the approved institutional protocol (2016N000136). Before injection of DFO‐NP and DFO‐NP loaded hydrogel formulations, six‐week‐old CD‐1 mice (male; 25–30 g) from Charles River Laboratories (Wilmington, MA) were anesthetized with isoflurane and oxygen, and blood was sampled in capillary tubes (Fisher Scientific, Pittsburgh, PA) at time point 0 min by slightly cutting the end of the tail. For subcutaneous injection, DFO‐NP was dissolved in DW at a concentration of 30 wt%, and DFO‐NP/F127, DFO‐NP/HA, and DFO‐NP/HA/F127 were prepared as described above. The mice were separated into 4 groups (*n* = 3). 100 µL of each formulation was injected subcutaneously into the back of mice. After injection, in vivo fluorescence images were taken using the NIR imaging system (K‐FLARE) with an 800 nm channel at predetermined time points and blood samples were collected using capillary tubes at the same time. The fluorescence intensities of serum samples in capillary tubes were measured with Cytation5. 14 d after injection, mice were sacrificed to observe the biodistribution of DFO‐NP and the organs (liver, lung, spleen, kidney, intestine, and bladder).

### Iron chelation efficacy of DFO‐NP/HA/F127

CD‐1 mice (male; 30–35 g; Charles River Laboratories) were fed a high‐iron diet (10 000 ppm Fe per kg) for 1 week. After, all mice were treated with facility chow (300 ppm Fe per kg) and administered saline, blank NP/ HA/F127 (84 µmol kg^−1^ as NP), or DFO‐NP/HA/F127 (84 µmol kg^−1^ as NP) by SC injection. At 3 weeks post administration, mice were euthanized, followed by heart, liver, and spleen collection for the analysis of iron contents by a nonheme iron colorimetric analysis using bathophenanthroline disulfonic acid.

### Histological Analysis of Organs

Resected organs (heart, lung, liver, spleen, and kidney) were stored at −80 °C. The frozen organs were fixed in 10% neutral buffered formalin and were dehydrated in ethanol, embedded in paraffin, and sectioned into slices (5 µm). Then, those sections were stained with hematoxylin and eosin (H&E) for pathology observation under the optical microscopic system (Cytation 5, BioTek Instruments).

### Toxicity Study

To evaluate the toxicity of DFO‐NP and DFO‐NP loaded hydrogel formulations, blood samples were collected by cardiac puncture 14 d after injection. These blood samples were stored at room temperature without anticoagulant for 30 min and were then centrifuged at 3000 rpm for 15 min. Serum was stored at −80 °C until further assays. Activities of aspartate aminotransferase (AST) and alanine aminotransferase (ALT) were measured for hepatotoxicity, and blood urea nitrogen (BUN) and creatinine (CREA) were measured for nephrotoxicity. All assays were performed using commercially available assay kits and the absorbance was immediately measured by a plate reader.

### Statistical Analysis

The fluorescence and background intensities of a region of interest over each organ were quantified using customized imaging software and ImageJ v1.52i (National Institutes of Health, Bethesda, MD). The signal‐to‐background ratio (SBR) was calculated as SBR = fluorescence/background, where the background is the fluorescence intensity of muscle or system background. Data are presented as mean ± s.e.m. with a minimum of three replicates. Student's *t*‐test statistical analysis was performed to evaluate the significance of the experimental data. The differences among groups were determined using one‐way ANOVA analysis with Bonferroni's multiple comparison corrections to assess the statistical differences among more than two groups. *p* values of less than 0.05 were considered significant. The data was indicated with *p < 0.05, **p < 0.01, and ***p < 0.001.

## Conflict of Interest

The authors declare no conflict of interest.

## Author Contributions

S.H.P., H.K., and H.S.C. conceived the study and designed the experiments. S.H.P., R.S.K. W.R.S., M.J., S.R., Y.B., and H.K. performed the experiments and analyzed the results. S.H.P., J.K., M.S.K., H.K., and H.S.C. interpreted the data and wrote the manuscript. All authors discussed the results and commented on the manuscript.

## Supporting information

Supporting Information

## Data Availability

The data that support the findings of this study are available in the supplementary material of this article.
