# Supplementary material for: Injectable Thermosensitive Hydrogels for a Sustained Release of Iron Nanochelators
Source: Adv Sci (Weinh). 2022 Mar 27;9(15):2200872. doi: 10.1002/advs.202200872 (PMC9130884; doi:10.1002/advs.202200872)
Supplement: Supplementary file 1 — Supporting Information [file ADVS-9-2200872-s001.pdf]

ADVANCED SCIENCE Open Access

Supporting Information

for *Adv. Sci.*, DOI 10.1002/advs.202200872

Injectable Thermosensitive Hydrogels for a Sustained Release of Iron Nanochelators

Seung Hun Park, Richard S. Kim, Wesley R. Stiles, Minjoo Jo, Lingxue Zeng, Sunghoon Rho, Yoonji Baek, Jonghan Kim, Moon Suk Kim, Homan Kang* and Hak Soo Choi**

Supporting Information

Injectable thermosensitive hydrogels for a sustained release of iron nanochelators

Seung Hun Park[1,2], Richard S. Kim[1], Wesly R. Stiles[1], Minjoo Jo[1], Sunghoon Rho[1], Yoonji Baek[1], Jonghan Kim[3], Moon Suk Kim[2*], Homan Kang[1,*], and Hak Soo Choi[1,*]

[1]Gordon Center for Medical Imaging, Department of Radiology, Massachusetts General Hospital and Harvard Medical School, Boston, MA 02114, United States
[2]Department of Molecular Science and Technology, Ajou University, Suwon16499, South Korea
[3]Department of Biomedical & Nutritional Sciences, Zuckerberg College of Health Sciences, University of Massachusetts, Lowell, MA 01854, United States

*Correspondence to H.K. at hkang7@mgh.harvard.edu; M.S.K. at moonskim@ajou.ac.kr; or H.S.C. at hchoi12@mgh.harvard.edu

The file includes:

Supplementary Methods

Supplementary Tables and Figures

Table S1. Summary of current iron chelation agents.

Figure S1. Synthetic routes of DFO-NPs

Figure S2. ^{1}H-NMR spectra of DFO, ZW-EPL$^+$, ZW-EPL$^-$, and DFO-NP

Figure S3. Hydrodynamic diameter (HD) analysis of DFO-NPs

Figure S4. Optophysical properties of DFO-NPs

Figure S5. *In vitro* cellular uptake and viability studies for DFO-NPs

Figure S6. Synthetic routes of crosslinked HA (xHA)

Figure S7. Biodistribution of DFO-NPs at 14 d post-injection

Figure S8. Representative fluorescence images of blood samples in capillary tubes

Figure S9. H&E staining images of heart, lung, liver, spleen, and kidney in saline

Supplementary Methods

Synthesis of ZW800-1C conjugated EPL (ZW-EPL$^+$): To prepare ZW800-1C conjugated EPL. 1 g of epsilon poly-L-lysine (EPL) was dissolved in 100 mL of phosphate buffer saline. The pH of the EPL solution was adjusted to around 8.0 with 6 M NaOH solution. ZW800-1C-succinic amide ester (ZW800-1C-NHS) was dissolved in DMSO at a concentration of 25 mg mL^{-1}. 5 mL of ZW800-1C-NHS solution was dropped into the EPL solution with vigorous stirring at room temperature. After 3 hours, the reaction mixture was added into 1 L of acetone/ethyl acetate (EA) (4/1) to precipitate ZW800-EPL. The mixture was centrifuged at 3000 rpm for 15 min at 4°C. The supernatant was discarded, and the precipitant was redissolved in DW (>50 mL) and re-precipitated in 1 L of acetone/EA. The precipitation was repeated two more times to complete the purification. The final precipitant was dried in a vacuum overnight.

Synthesis of succinylated ZW800-EPL (ZW-EPL$^-$): ZW800-EPL$^+$ (1g, 0.2 mmol) and EPL (19 g, 4.75 mmol) were dissolved together in 2 L of PBS. 45 g of succinic acid (450 mmol) in 180 mL of DMSO (250 mg/ mL) was added to the ZW-EPL solution. The pH of the reaction mixture was adjusted to around 7.0 with 6 M NaOH solution as needed. The reaction mixture was stirred for 30 min at room temperature. After, the succinylation ratio was confirmed by the ninhydrin test. For purification, the reaction mixture was precipitated with the same procedure described in the previous section.

Synthesis of renal clearable nanochelator (DFO-NP): 50 g of deferoxamine (76 mmol) was dissolved in 500 mL of DW. The DFO solution was neutralized by adding of 6 M NaOH solution. 20 g of ZW-EPL$^-$ (2.5 mmol) was dissolved in 1.25 L of DW. 42 g of DMTMM and the prepared DFO solution were added to the ZW-EPL$^-$ solution. The reaction mixture was stirred for 2 h at 60°C, followed by dialysis performed against DW with a cellulose dialysis membrane of MWCO 6-8 kDa. After dialysis, the solution was lyophilized.

Size-exclusion chromatography analysis: To measure the purity and hydrodynamic diameter (HD) of DFO-NP, size-exclusion chromatography (SEC) was performed with the Waters HPLC system consisting of a Waters e2695 separations module and Waters 2998 PDA detector. The column used was an Xbridge BEH 125Å 3.5 µm (7.8 x 150 mm, Waters) SEC column. The mobile phase was isocratic with 10 mM PBS for 15 min at a flow rate of 0.75

mL min^{-1}. Each component in the reaction mixture could be identified by its retention time and absorbance wavelength.

The standard calibration curve of HD was calculated by injecting 10 µl of protein standards containing aprotinin (6.5 kDa, 1.96 nm), ribonuclease (13.7 kDa, 3.28 nm), ovalbumin (44 kDa, 6.10 nm), and thyroglobulin (669 kDa, 9.6 nm) to the HPLC with the same mobile phase and flow rate described above. The partition coefficient, K_{av} was obtained from the following equation (1):

$$K_{av}\ (V_e - V_0)/(V_c - V_0) \qquad (1)$$

, where V_0, V_c, and V_e are column void volume, geometric column volume, and eluent volume, respectively. The HD of DFO-NP was calculated by the following equation (2):

$$HD = 3.778\left(\frac{0.4608 - K_{av}}{K_{av} + 0.008565}\right)^{\frac{1}{2.65}} \qquad (2)$$

^{1}H-NMR analysis: To determine the DFO conjugation ratio on the nanochelator, 10 mg of DFO-NP was dissolved in 600 µL of D_2O. ^{1}H-NMR spectroscopy was performed with a Varian 500 MHz spectrometer. The number of DFOs on succinylated EPL was calculated by comparing the peak integration values of succinylated EPL's protons at 4.1 ppm and DFO's position e,l,s protons at 3.6 ppm (**Figure S2**).

Optical properties of DFO-NP: DFO-NP was dissolved in DW at a concentration of 100 µM. Absorbance and fluorescence emission spectra were observed from 500 nm to 1000 nm with a UV/Vis/NIR spectrometer (USB2000, Ocean Insight, Dunedin, FL). For the fluorescence emission spectrum, a 760 nm laser (Nawoo, Gwangju, Korea) was used for excitation.

In vitro iron-chelating effect of DFO-NP: To confirm the iron-chelating effect of DFO-NP, a solution of DFO-NP was prepared at a concentration of 100 µM. 5 µL of ferric chloride solution (4 mM) was added to the DFO-NP solution with continuous measurement of the absorbance change at 430 nm which is the absorption value of the Fe^{3+} and DFO complex. The functional stoichiometry was calculated based on the titration curve prepared by using the absorbance change at 430 nm.

Cell culture: NIH3T3 and H23 cells were cultured in Dulbecco's modified eagle medium (DMEM, Mediatech, Herndon, VA) that contained 10% fetal bovine serum and 1% penicillin-streptomycin. NIH3T3 and H23 were incubated in a 75 cm^2 tissue culture flask (Corning, NY) under 5% CO_2 at 37°C, respectively.

In vitro cellular uptake and cytotoxicity of DFO-NP: NIH3T3 (1×10^4) and H23 (1×10^4) cells were seeded in a 96-well plate (Corning, NY) and cultured for 24h, respectively. Cells were washed with 200 µL of PBS two times. 100 µL of fresh complete medium containing ZW800-EPL+ (5 uM) or DFO-NP (5 uM) was added to each well. Cells were incubated for 30 min and 2 h. The cells without any treatments were used as a control. At predetermined time points, cells were washed with PBS, followed by being fixed with 4% paraformaldehyde. The fixed cells were observed using Cytation5 (BioTek, Winooski, VT) and NanoenTek JuLi Stage (Seoul, S. Korea) in a bright and NIR fluorescence channel.

For the cytotoxicity test, NIH3T3 cells were treated with DFO-NP ranging from 1 to 100 µM. After 24h, NIH3T3 cells were washed with PBS twice. 100 µL of fresh complete medium was added to each well. Then, 10 µL of CCK8 solution was added to each well. After 4 h, the absorbance was measured at 450 nm using a microplate reader (Cytation5). All experiments were carried out with three replicates.

Table S1. Summary of current iron chelation agents.

Name	Type	Half-life	Status	Ref.
Deferoxamine (DFO)	Small molecule	< 1h	Marketed	[6]
Deferasirox (DFX)	Small molecule	8-16 h	Marketed	[7]
Deferiprone (DFP)	Small molecule	2-3 h	Marketed	[8]
desferrithiocin	Small molecule	N/R	Preclinical[a]	[8]
Triapine	Small molecule	4-5 h	Phase II	[8]
di-2-pyridyl ketone thiosemicarbazone (Dpc)	Small molecule	11.7 h	Phase I	[8]
ROS-responsive polymeric nanogels containing DFO (rNG-DFO)	Nanogel	N/R	Preclinical	[9]
Oxidation-sensitive iron chelating nanogels (oxNG–DFO)	Nanogel	N/R	Preclinical	[9]
PEG-functionalized melanin nanoparticles (PEG-MNP)	Nanoparticle	17 h	Preclinical	[9]
Ultra-long circulating DFO (ULC-DFO)	Dendrimer	44 h	Preclinical	[9]
Dendritic polyglycerol- DFO (HPG–DFO)	Dendrimer	16 h	Preclinical	[9]
Polyethylene glycol conjugated (P-DFO)	Macromolecule	N/P	Preclinical	[9]
Dextran-DFO (DEX-DFO)	Macromolecule	1 h	Preclinical	[9]
Pentastarch-DFO (MPS-DFO)	Macromolecule	1-2 h	Preclinical	[9]
Hydroxyethyl starch –DFO (HES-DFO)	Macromolecule	1.4 h	Preclinical	[9]
Hyper branched polyglycerol conjugated DFO (BHPG-DFO)	Dendrimer	8 h	Preclinical	[9]
ε-Polylysine-DFO (DFO-NP)[b]	Macromolecule	1 h	Preclinical	[9]

[a]The chelator is known to be nephrotoxic. [b]The renal clearable nanochelator, DFO-NP, is used in this study. [c]N/R: not reported.

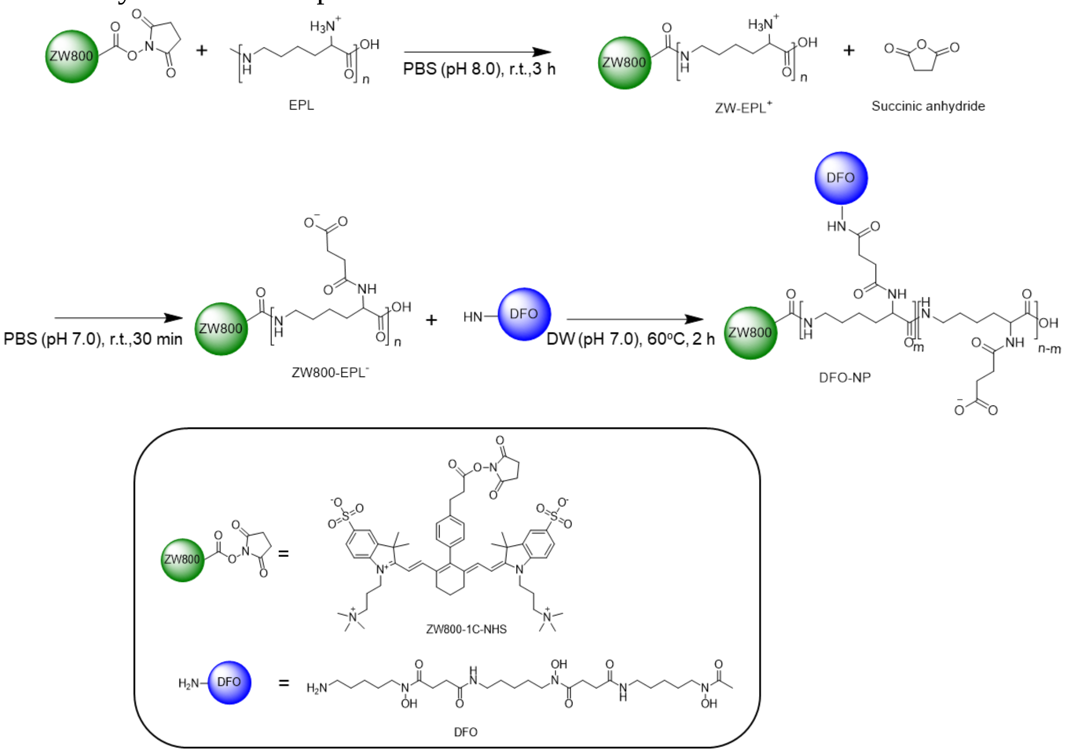

Figure S1. Synthetic route of DFO-NP.

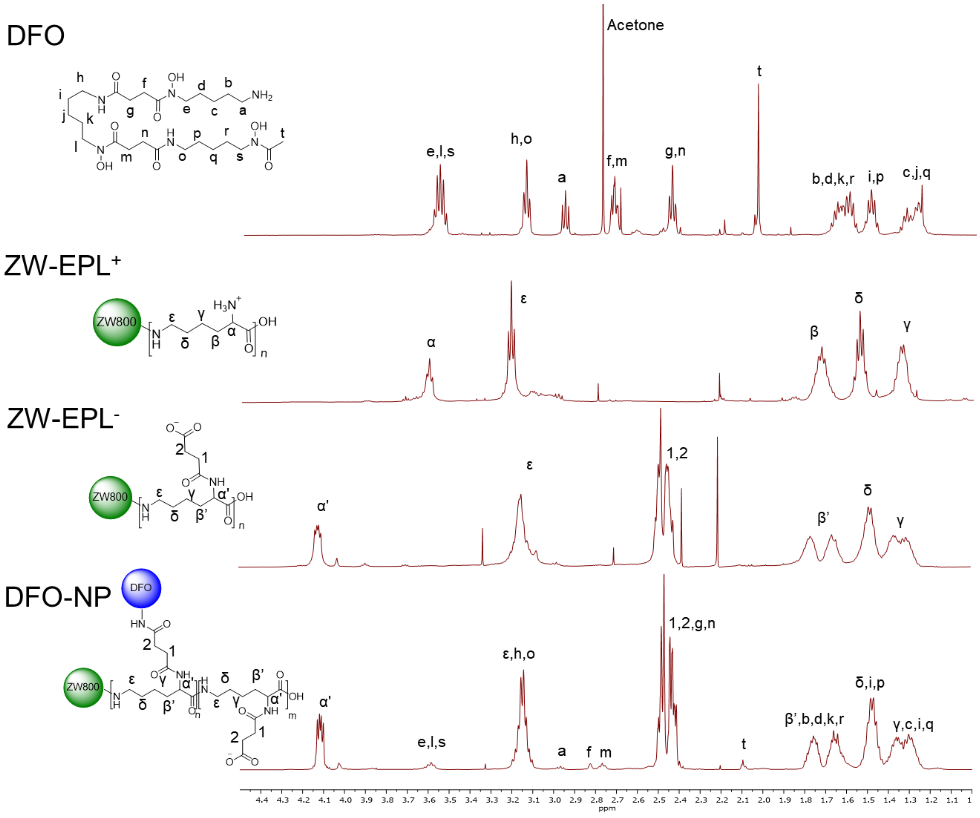

Figure S2. ^{1}H-NMR spectra of DFO, ZW-EPL$^+$, ZW-EPL$^-$, and DFO-NP. DFO to ZW-EPL$^-$ ratio was calculated by peak integration of the ZW-EPL$^-$ protons (positions α' at 4.12 ppm),

and DFO protons (position e,l,s at 3.6 ppm). The calculated values of DFO per chain were based on the assumption of 30 lysine units per entire ZW-EPL^{-}.

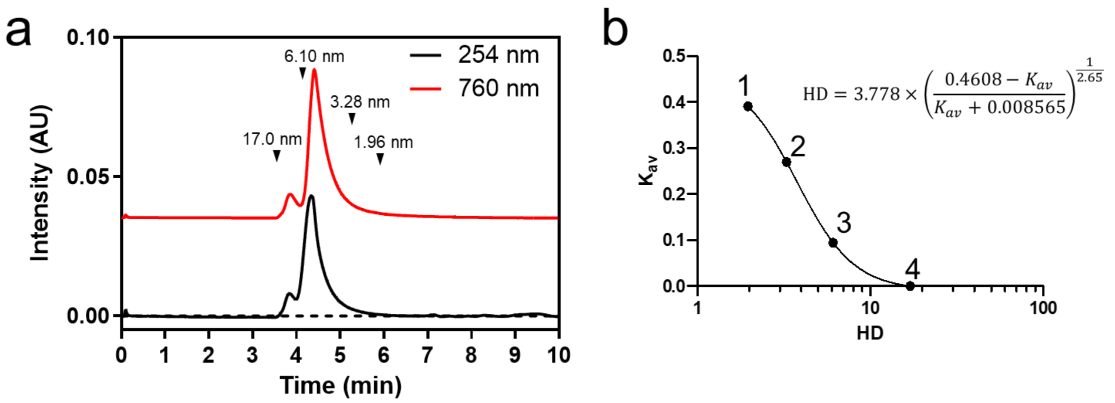

Figure S3. Hydrodynamic diameter (HD) analysis of DFO-NPs. A) Size-exclusive chromatography measured at 254 nm (black line) and 760 nm (red line), respectively. Arrowheads and numbers indicate the corresponding HD of standard proteins: Aprotinin, 1.96 nm; ribonuclease, 3.28 nm; ovalbumin, 6.10 nm; thyroglobulin, 17.00 nm. B) The standard calibration curve of HD using the standard proteins.

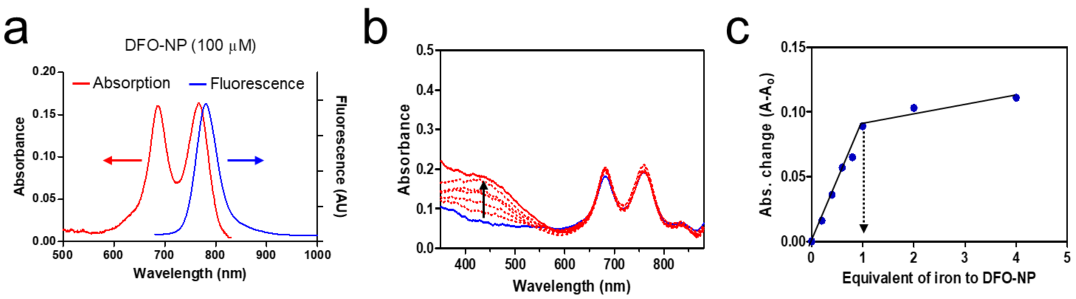

Figure S4. Optophysical properties of DFO-NPs. A) Optical properties including absorption and fluorescence spectra at a concentration of 100 μM. B) UV-vis absorption spectra of Fe(III)-titrated DFO-NP (blue line indicates DFO-NP solution before adding iron solution, red lines indicate after titration after titration with the iron solution. C) A titration curve of absorbance change (A-A_0).

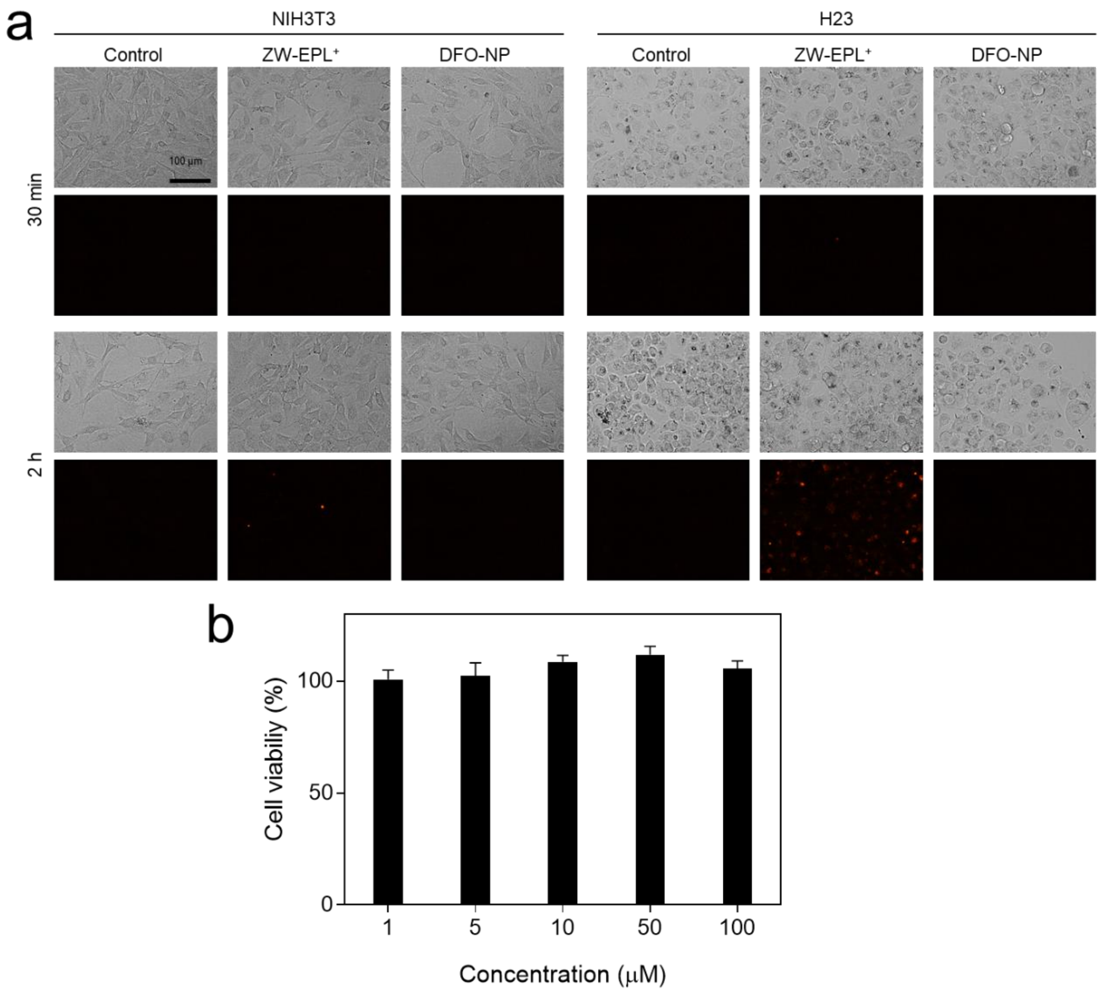

Figure S5. In vitro cellular uptake and viability tests for DFO-NPs. A) Cellular uptake study for ZW-EPL$^+$ (5 μM) and DFO-NP (5 μM) on NIH3T3 and H23 cells. B) Cell viability of NIH3T3 cell at various concentrations of DFO-NP.

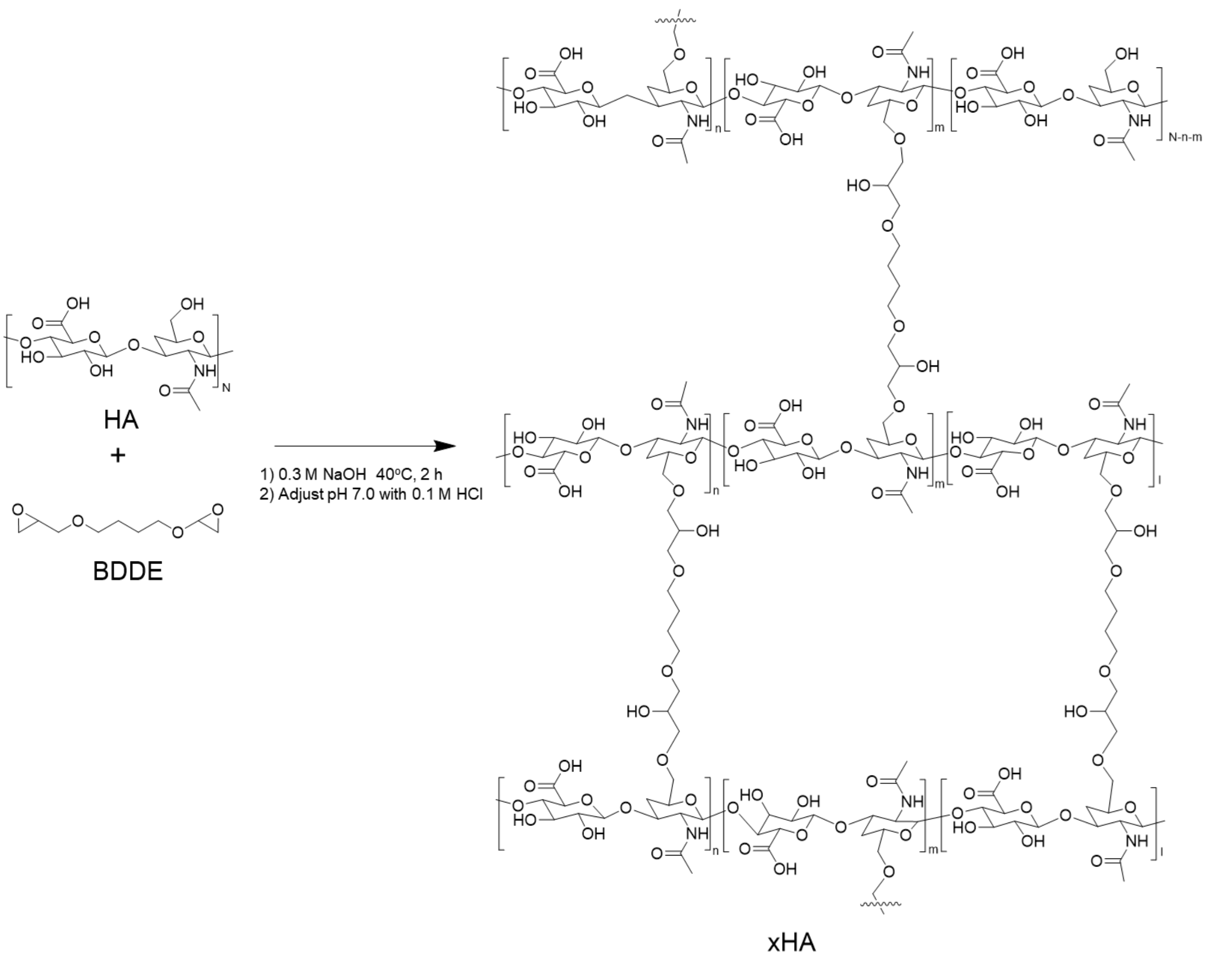

Figure S6. The synthetic scheme of crosslinked HA (xHA).

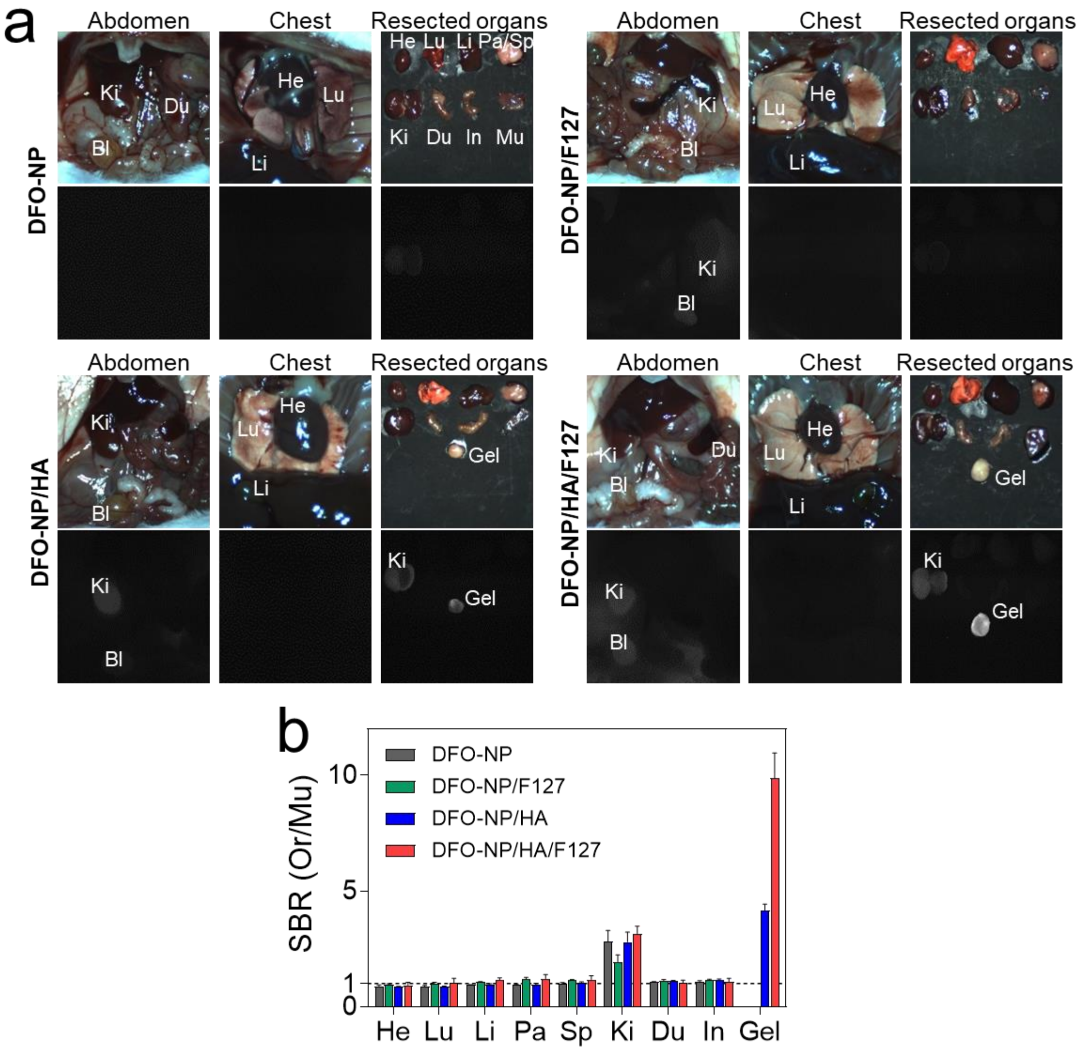

Figure S7. Biodistribution of DFO-NPs at 14 d post-injection. A) Color and NIR fluorescence images of abdomen, chest, and resected organs of mice subcutaneously injected with DFO-NP and DFO-NP loaded hydrogel formulations. B) Signal-to-background ratio (SBR) of resected organs and hydrogel against muscle. He, heart; Lu, lung; Li, liver; Pa/Sp, pancreas/spleen; Ki, kidney; Du, duodenum; In, intestine; Mu, muscle

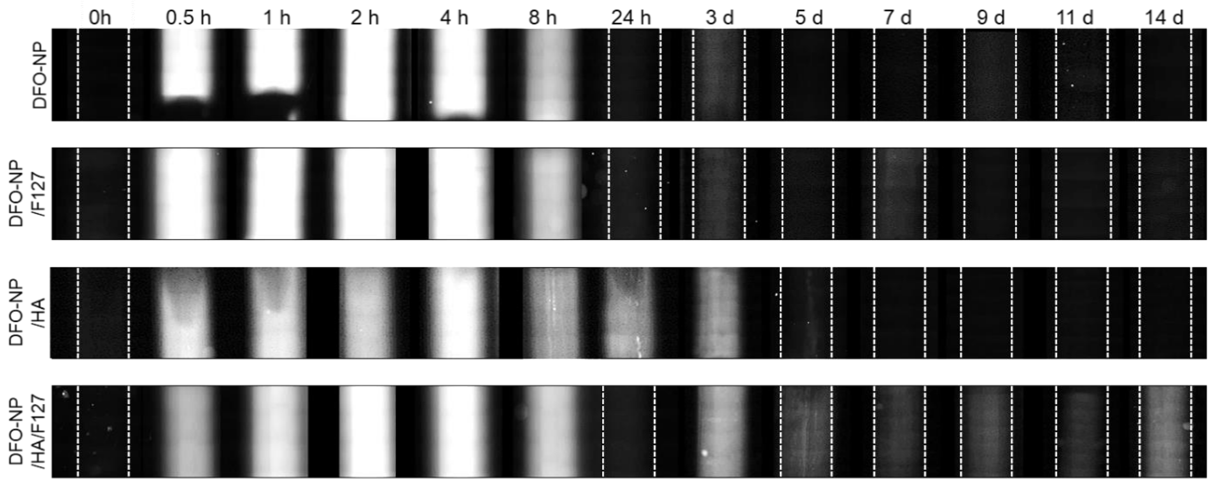

Figure S8. Representative fluorescence images of blood samples in capillary tubes at each time point.

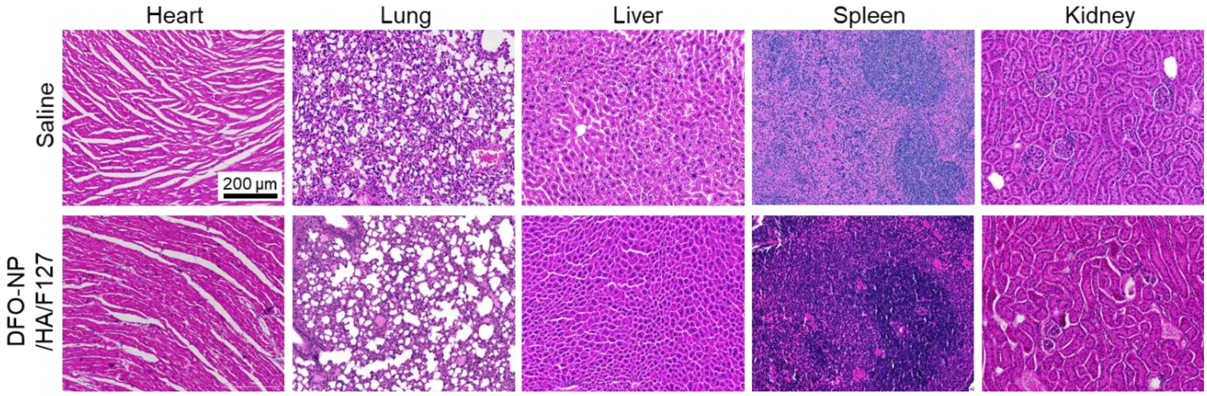

Figure S9. H&E staining images (10×) of heart, lung, liver, spleen, and kidney in saline and DFO-NP/HA/F127 groups.
